# Neonatal non-contact respiratory monitoring based on real-time infrared thermography

**DOI:** 10.1186/1475-925X-10-93

**Published:** 2011-10-20

**Authors:** Abbas K Abbas, Konrad Heimann, Katrin Jergus, Thorsten Orlikowsky, Steffen Leonhardt

**Affiliations:** 1 Philips Chair for Medical Information Technology, RWTH Aachen University, Pauwelsstr. 20, 52074 Aachen, Germany; 2 Department of Neonatology, RWTH Aachen University Hospital, Pauwelsstr. 30, 52074 Aachen, Germany

## Abstract

**Background:**

Monitoring of vital parameters is an important topic in neonatal daily care. Progress in computational intelligence and medical sensors has facilitated the development of smart bedside monitors that can integrate multiple parameters into a single monitoring system. This paper describes non-contact monitoring of neonatal vital signals based on infrared thermography as a new biomedical engineering application. One signal of clinical interest is the spontaneous respiration rate of the neonate. It will be shown that the respiration rate of neonates can be monitored based on analysis of the anterior naris (nostrils) temperature profile associated with the inspiration and expiration phases successively.

**Objective:**

The aim of this study is to develop and investigate a new non-contact respiration monitoring modality for neonatal intensive care unit (NICU) using infrared thermography imaging. This development includes subsequent image processing (region of interest (ROI) detection) and optimization. Moreover, it includes further optimization of this non-contact respiration monitoring to be considered as physiological measurement inside NICU wards.

**Results:**

Continuous wavelet transformation based on Debauches wavelet function was applied to detect the breathing signal within an image stream. Respiration was successfully monitored based on a 0.3°C to 0.5°C temperature difference between the inspiration and expiration phases.

**Conclusions:**

Although this method has been applied to adults before, this is the first time it was used in a newborn infant population inside the neonatal intensive care unit (NICU). The promising results suggest to include this technology into advanced NICU monitors.

## Introduction

Basically, vital signals are physical quantities measured from the body and can be used to determine the physiological status and functioning. Examples of these signals include heart rate, breathing rate, body temperature and blood pressure. The normal range of vital signs varies with age, sex, weight, exercise tolerance and body conditions [1,2]. Nasal inspiration, the way neonates acquire air and hence oxygen, is important for maintaining the internal milieu of the lung, since ambient air is conditioned to nearly alveolar conditions (i.e. body temperature and fully saturated with water vapor) upon reaching the nasopharynx cavity. Essentially, respiration measurement can be performed by using nasal thermocouples, respiratory-effort belt transducer, piezoelectric transducer, optical sensor (pulse oximetry) and electrocardiography ECG. However, all these techniques are inconvenient to take in at home and they may bring discomfort and soreness to the patient [2-4]. Apnoea (abrupt stopping of respiration) and bradycardia (rapid decrease of heart rate) are common and serious problems in premature infants. One of the methods to quantify respiratory rate in these infants is to use a thermistor that is fixed above the upper lip directly in front of the nares. This by itself can induce apnoeas because of upper respiratory airway obstruction. Therefore, one of important field in such monitoring system is neonatal intensive care unit (NICU), where the patients (neonates) need continuous monitoring of such vital signs (e.g. respiration rate) without creating a discomfort or irritation to them. In principle, optical, electromagnetic, acoustic, and pneumatic techniques can be employed to realize noncontact measurement of physiological quantities. Wang et al. [2] performed a study on non-contact detection of breathing and heart beat based on radar principles. Similarly, Droitcour et al. [5] developed a respiratory rate monitoring system using a non-contact, low power 2.4 GHz Doppler radar system and obtained good results when monitoring breathing activities for hospitalized patients. De Chazal et al. [3] modified a biomotion sensing technique for respiratory activity detection based on 5.8 GHz Doppler radar. Hafner et al. [6] developed non-contact cardiopulmonary sensing with a baby monitor for premature infants inside neonatal intensive care unit (NICU) by using simple Doppler radars operating in continous wave (CW) mode. Moreover, Zito et al. [7] developed a wearable system-on-chip (SoC) ultra wide band (UWB) radar for contactless cardiopulmonary monitoring. Matusi [8] has proposed a novel approach for touchless measurement of heart rate variability (HRV) by using a combination of microwave radar and infrared thermography to analyze the exhaled CO/CO_2 _gas concentrations. Furthermore, Mathews et al. [4] also prototyped a contactless vital signal monitor which uses very low power, high frequency Doppler radar to detect the respiration and heart rates. Ling et al. [9] introduced the OxyArm module, which is a new minimal contact oxygen delivering system for mouth or nose breathing. Moreover, Hoffmann et al. [10] developed a capacitive textile force sensor for detecting respiration activity rate in the human body. Additionally, Nakajim et al. [11] employed a real-time image sequence analysis of CCD video camera for evaluating posture changes and respiratory rate of a subject in bed. Moreover, Heimann et al. [12] investigate a new non-contact monitoring method of heart and lung activity using magnetic induction measurement. In premature infants, a thermistor was used to quantify respiratory flow during inspiration and expiration with the disadvantage of a possible obstruction of the upper airways.

In contrast to the infrared (IR) detectors, which measure the radiation energy emitted from any object containing solid matter, that may represent an option for passive non-contact measurement of vital signs including respiration activity [13]. Resume to that, the skin is the largest organ of the human body and helps maintain the thermal equilibrium of the body and the environment through a heat transfer process.

### Infrared thermographic imaging

**Figure 1:**
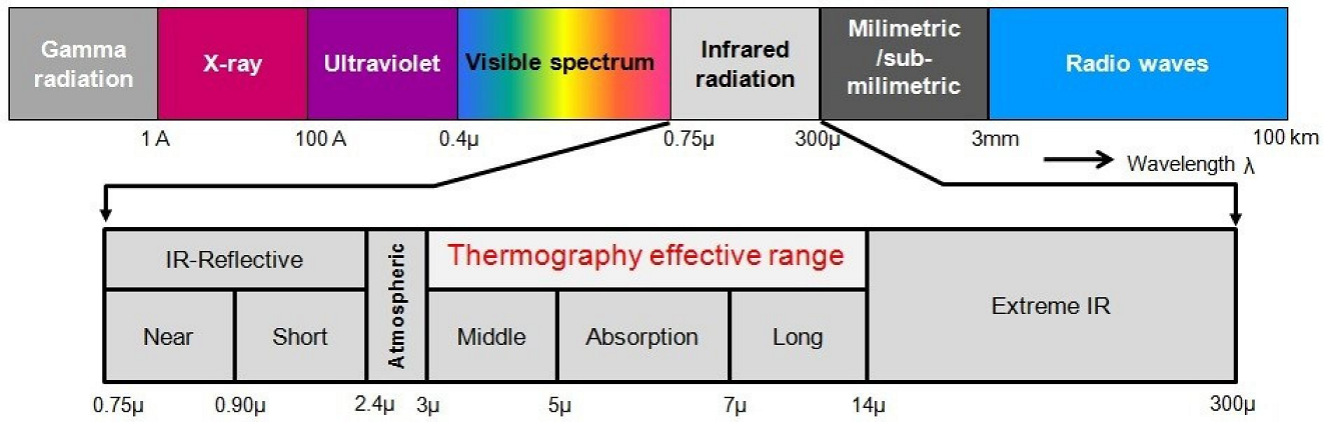
**The electromagnetic spectrum**. The electromagnetic spectrum showing the infrared radiation classification and corresponding wavelengths, (^© ^MedIT, 2011).

Any object whose temperature is above absolute zero Kelvin (-273.15°C) emits radiation at a particular rate and with a distribution of wavelengths. This wavelength (λ) distribution is dependent on the temperature of the object and its spectral emissivity ϵ(λ). The spectral emissivity, which can also be considered as the radiation efficiency at a given wavelength, is in turn characterized by the radiation emission efficiency based on whether the body is a black body, grey body, or a selective radiator. Around room temperature, the typical emissions for a solid matter are maximal in the long wave infrared (LWIR) region of the electromagnetic spectrum (7 *μ*m to 14 *μ*m) (see Figure 1). In principle, the hotter the object, the higher its maximal frequency of radiation, that moves towards the visible region. While IR radiation is invisible to the human eye, it can be detected and visualized by special IR cameras. These cameras detect the invisible IR radiation emitted by an object and convert it to a monochrome or multicolored image on a monitor screen, in which the various shades or colors represent the thermal patterns across the object's surface [1,14]. In one specific medical application the thermal imagers may be coupled with proper computer software to detect febrile temperatures on the skin of passengers (epidemic screening). Temperature readings over time may also be registered. Although thermal imagers offer an excellent means of making a qualitative determination of surface temperature, there are difficulties in obtaining absolute measurements.

### Target's surface radiation heat exchange

**Figure 2:**
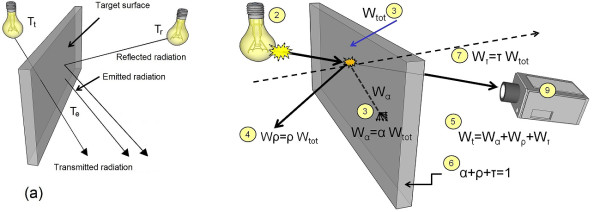
**Heat radiation mechanism**. Heat radiation mechanism (a) The radiative heat flow mechanism, where total exitance or radiosity equal to the sum of reflected radiation $W_{r}=\varepsilon .\sigma .T_{r}^{4}$, transmitted radiation $W_{t}=\varepsilon .\sigma .T_{t}^{4}$ and emitted radiation $W_{e}=\varepsilon .\sigma .T_{e}^{4}$, (b) The concept of impinging the process of radiation on a target surface, where (1) is the total radiant energy, (2) heat source,(3) absorbed energy, (4) reflected energy, (5) transmitted energy, (6) total detected energy and (7) total surface properties (^© ^MedIT, 2011).

The measurement of IR thermal radiation is the basis for non-contact temperature measurement and thermography. Fundamentally, thermal IR radiation (W) leaving a surface (A) is called 'exitance' or 'radiosity'. This energy W can either be emitted from the surface, reflected off the surface, or transmitted through the surface (see Figure 2) [14,15]. Note that the total radiosity is equal to the sum of the emitted component (W_*e*_), the reflected component (W_*r*_) and the transmitted component (W_*t*_). Thus, the surface temperature is related to W_*e*_, only on the emitted component [15,16]. Generally, the infrared radiation impinging on the surface can be absorbed, reflected or transmitted, as shown in Figure 2(b). Basically, Kirchhoff's law of thermal radiation states that the sum of the three components is always equal to the received radiation. Hence, the sum of the three components, if the percentages are expressed as fractions, equals unity

(1) α(absorptivity)+ρ(Reflectivity)+τ(transmittance)=1

Therefore, each part of these physical quantities has a value equal to less than one, as can be seen in the three radiation components in Figure 2(a). For further simplification, if the IR thermal energy detector is positioned in front of the target surface, then the net radiation (W_*net*_) which will be detected is equal to the transmitted component of the IR thermal radiation (see Figure 2(b)) [1,13,17].

(2) $$W_{net}=W_{\tau }=\tau \cdot W_{tot}$$

This means that the other two physical quantities (absorbed and reflected radiation) will not be considered in IR thermal imaging.

## Material and Methods

### Clinical Acquisition of IR Thermograms

**Figure 3:**
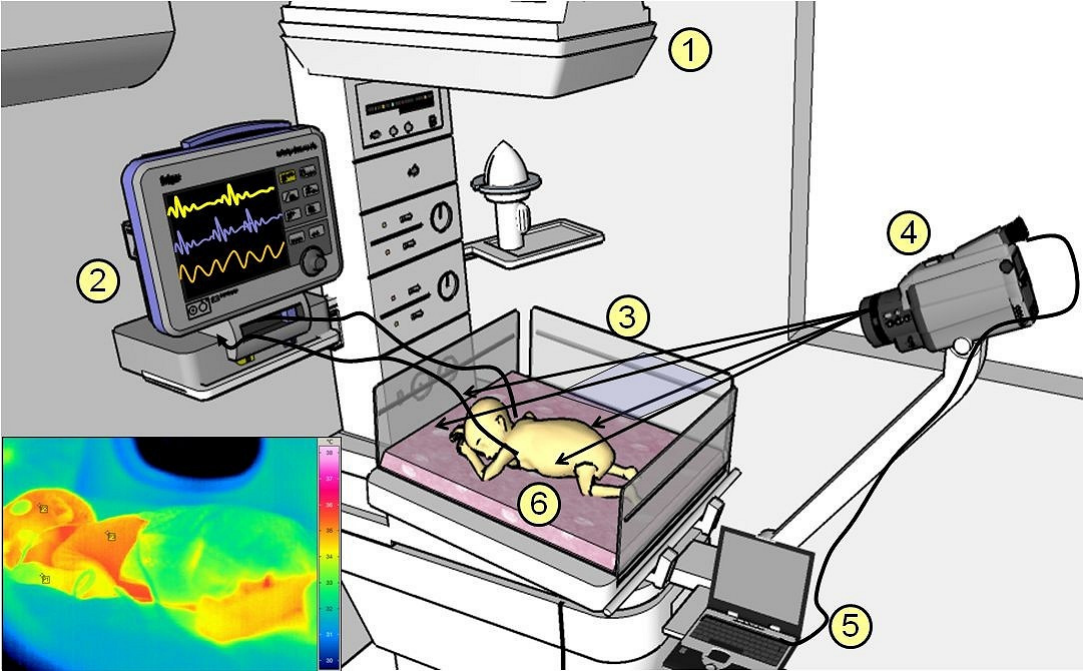
**IRTR clinical imaging setup**. Schematic of the experimental setup used for the neonatal infrared respiration monitoring technique. The IR camera is located 70 - 80 cm from the neonate and is connected to the IR acquisition/analysis workstation. The infant's nostrils have to be in direct optical contact and visible, the overall setting consist of (1) radiant warmer bed,(2) bedside monitor,(3) camera field of view (FOV),(4) IR thermal camera, (5) analysis workstation and (6) infant under NIRT imaging (^© ^MedIT, 2011).

**Figure 4:**
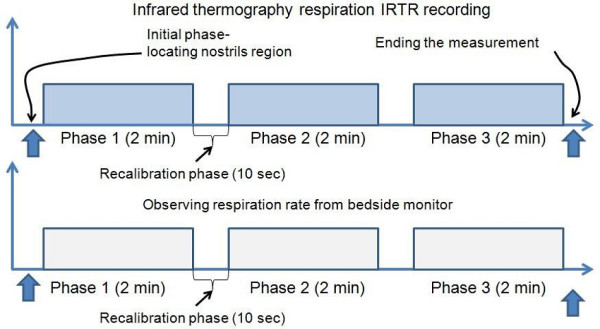
**IRTR measurement protocol**. The IRTR signal measurement protocol which include three phases and in between a recalibration phase to compensate any non-uniformities within infrared thermography imaging.

The real-time IR thermograms were collected using a VarioCAM^® ^hr head IR camera (InfraTec GmbH, Germany) with a thermal sensitivity of 0.05°C at 30°C. This device allows IR image transfers via the IEEE-1394 Firewire data interface at 30 frame per second (fps) frame rate. The scaling temperature sensitivity scheme in the infrared radiation range of 1 *μ*m to 14 *μ*m was set to a range of 0°C to 40°C. Preprocessing steps (such as filtering, color scale conversion and image scaling) were done using IRBIS^® ^professional software. Figure 3 shows the imaging setup for neonatal thermal respiration (IRTR) measurement. The acquisition protocol for the IRTR measurement consists of three distinct phases with 2 minute duration for each phase, and between these phases intervals, there is a recalibration time to correct any non-uniformity with IR themrography (IRT) (see Figure 4). Note that, the observation phases from the bedside monitor have the same phases. Furthermore, at the initial measurement time, a localizing of neonate's nostrils region to make sure that full coverage of IRTR signature is performed.

### Patient population

All measurements were conducted at the Department of Neonatology (RWTH Aachen University Hospital). This has been approved by the Medical Ethics committee of the RWTH Aachen University Hospital, issued on 19 August 2009 (EK032/09). We examined seven premature infants with a median gestational age of 29 weeks. They were all consecutively admitted directly after birth to our Department of Neonatology. We excluded infants with additional risk factors apart from prematurity, e.g. chromosomal abnormalities or brain haemorrhage. Study design and protocol were approved by the Ethics Committee of Aachen University Hospital and parental consent was obtained prior to enrollment. None of the infants was mechanically ventilated. They all had respiratory support via CPAP (Continuous Positive Airway Pressure) directly after birth because of respiratory distress syndrome, a very common disease in these infants. One of them still had a CPAP during the study. While five infants were handled in an incubator, two infants were positioned in an IR radiant warmer bed. Cardiorespiratory stability was a precondition to be included into the study to get a reliable signal over the whole time period of the IRT. During IRT, vital parameters including oxygen saturation were continuously monitored to make sure that there were no negative side effects. The authors are aware that this setup and this heterogenous patient population can not be a basis for a rigid clinical validation study. Instead, with this paper we are presenting a method for automated data analysis which may eventually lead to a valid ventilation monitoring technique.

### IR thermography post-processing

The acquired thermographic images were exported to MATLAB^® ^for post-processing and pre-filtering. The mean value was removed by a moving average filter. Motion compensation was applied using the trend-remove function of the MATLAB System Identification toolbox. The distance between the camera and the subject was kept at less than 150 cm in order to attenuate background IR radiation from the surrounding objects, and to eliminate geometrically induced disturbances. Initially, raw thermal data was used to construct time-varying signals for each thermographic pixel in the area of interest in order to build temperature time profile. However, this approach makes the signal extremely noisy due to variation in the ambient temperature of the surrounding region of interest (ROI) [18-21]. In our work, the thermal images were examined at different points in time. All frames that carry information relevant to respiration in the ROI were selected. Following these steps, the time-varying signals from each point in the ROI were averaged and continuous wavelet transform (CWT) was applied. The resulting waveforms yield excellent results to identify infrared thermography respiration (IRTR) signals [22,23]. The preliminary tests were conducted in our neonatal intensive care unit (NICU) to identify IRTR signals in neonates; stability over the measurement time intervals has been proven.

### Respiration thermal signature

**Figure 5:**
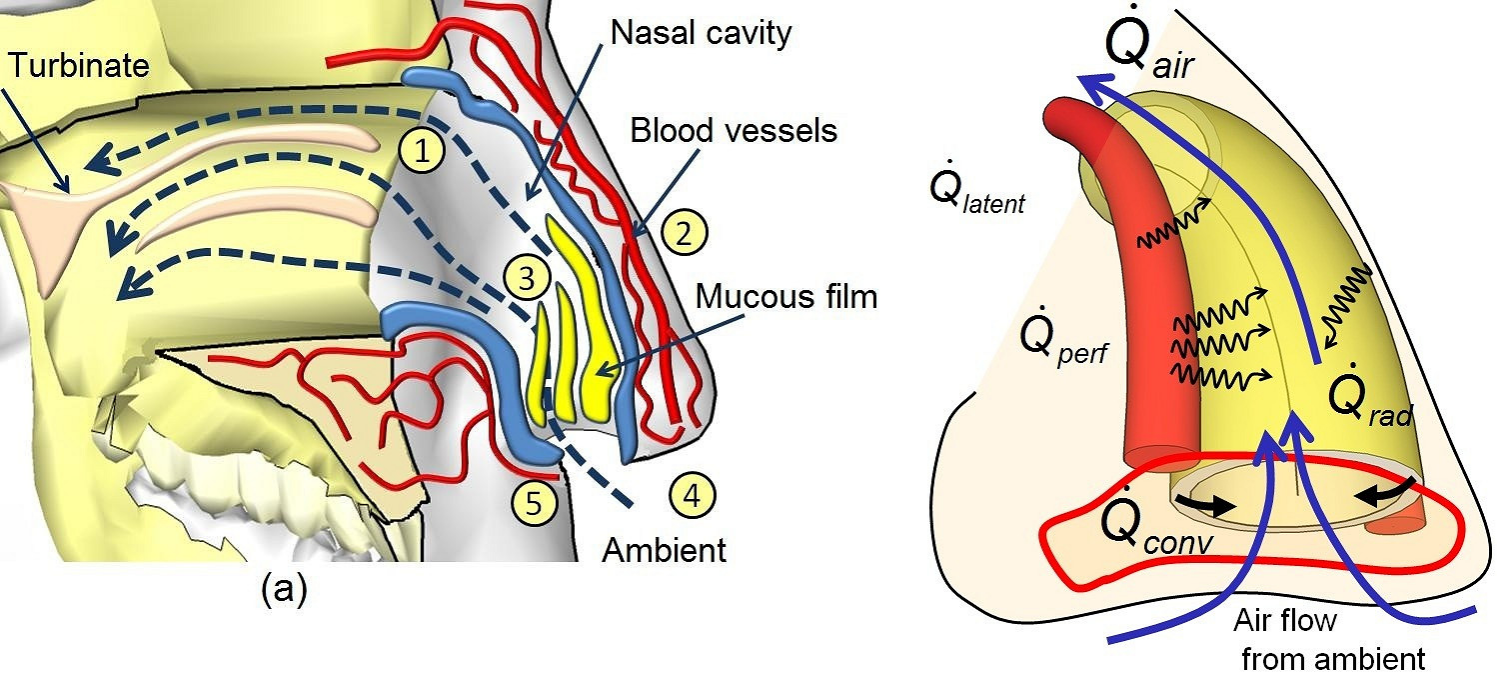
**Physiology of heat transfer processes inside nasal cavity**. (a) Anatomical section through the nasal cavity, showing the mechanism of heat exchange between the internal tissue lining and the flowing air inside the nasal cavity (inspiration-expiration phase) which consist of the following: (1) convective air flow inside nasal cavity, (2) perfusion heat transfer inside nasal blood vessels, (3) convective heat loss over mucosal film, (4) conductive heat loss of mucosal film on nostrils inner lining and (5) radiative heat loss from nostrils tissue, (b) schematic representation of heat transfer processes inside the nasal cavity, (^© ^MedIT, 2011).

Many physiological phenomena occur in the spectral band of long wave infrared (LWIR, 7 *μ*m to 14 *μ*m); however, in some bioheat transfer processes, these phenomena also take place in the range 3 *μ*m to 8 *μ*m mid-wave IR (MWIR) [13,14] or in the range of 0.7 *μ*m to 2 *μ*m short-wave infrared (SWIR) [15]. For example, Pavlidis et al. [1,20,24] used MWIR sensors for distant measurement of cardiac and breathing rates in adults. In this work, the breathing measurements were based on heat transfer of the moisturized air during expiration, which is directly related to the respiration waveform. Although the exact shape is smoothed, it was shifted and noisy with respect to the actual respiration rate. Most probably, this is mainly due to the diffusion-convection heat transfer processes and air flow in the nasal cavity [7,13,17]. Note that the anatomical section of a nasal cavity (shown in Figure 5) also consists of vascular mesh, which contributes to the temperature conditioning of the inhaled air. As indicated, for breathing measurements, Palvidis et al. [1,18,20,23,24] used the expired and moisturized air flow to measure the respiration rate. As a result, the subject must have a sideview technique to the camera in order to visualize breathing-jet dynamics. Beside this side view orientation, Pavlidis also introduced the concept of nostrils tracking in adults [18,20,23]. Our method presented here is related to this concept, but differs in the spectral range. Also, signal processing was enhanced. While other groups [20,23] used MWIR, our group used LWIR thermography, which is more stable in detection of temperature variance within the thermographic scenario [13,15,22]. In general, LWIR cameras are typically preferred for imaging applications that require absolute or relative measurements of object irradiance or radiance because emitted energy dominates the total signal in the LWIR. In the MWIR, extreme care is required to ensure the radiometric accuracy of data. Thermal references may be used in the scene to provide a known temperature reference point or points to compensate for detector-to-detector variations in response and improve measurement accuracy. This temperature reference measurement in our case is not possible, because this will include invasive nasal thermocouple and may interfere with our thermography image reading. Moreover, besides a characterization of the respiratorial behaviour, we focused on temperature changes in the nasal region (nostrils) within the thermal image to get possibly information about the impact on thermoregulation of the infants. Additionally, the thermal imaging was performed using both frontal and lateral views, in order to follow the defined region over the nostrils.

### Infrared Thermography Respiration (IRTR) signature detection

**Figure 6:**
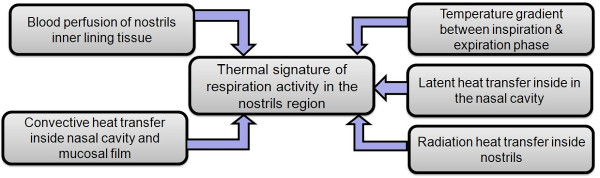
**Physical parameter interaction in respiration thermal signature**. Interaction of the physical parameters that contribute to the detection of the respiration thermal signature (^©^MedIT, 2011).

Basically, this type of thermographic imaging may be called "pulsed thermography", which implies that the process is repetitive in nature, which is true for the respiration rate. Therefore, it is essential to develop a method to detect a biphasic thermal breathing signal, which consists of two phases (active and passive states) [15,20,25]. Initially, during the application of infrared thermal imaging on newborn infants for mapping skin temperature, we were expecting to detect very small temperature changes in the nostrils region comparing to the magnitude in adults [20,23,26]. Therefore, we expected difficulties to detect the respiration thermal signature in infants, and were supposed to find this temperature difference between inspiration and expiration phase in the range between 0.3°C to 0.7°C. The physics of this phenomenon are based on the radiative and convective heat transfer component during the breathing cycle (see Figure 5). In fact, including all these influences in one model is a complex task, but should include the simulation of the airflow pathway, temperature gradient distribution throughout the nasal cavity, and the blood perfusion in the nasal cavity and nostril regions [18,25]. Hence, the mathematical approximation for the IRTR signature depends on the five main parameters shown in Figure 6, and the total heat flow rate $\left({\dot{Q}}_{RR}\left(t\right)\right)$ contributing to the thermal signature of one respiration cycle can be expressed as follows:

(3) $${\dot{Q}}_{RR}\left(t\right)={\dot{Q}}_{rad}\left(t\right)+{\dot{Q}}_{conv}\left(t\right)+{\dot{Q}}_{evap}\left(t\right)+{\dot{Q}}_{per\;f}\left(t\right)+{\dot{Q}}_{latent}\left(t\right)$$

The overall equation for heat transfer inside the nasal cavity at the nostril level will be equal to the summation of the following heat flow components:

• ${\dot{Q}}_{rad}$: rate of heat dissipated by radiation between the air flow and the nasal surface

• ${\dot{Q}}_{conv}$: rate of heat dissipated by convection between the nasal inner lining (skin and mucosa) and air flow

• ${\dot{Q}}_{evap}$: rate of heat dissipated by evaporation at the nasal surface (mucosal thin film)

• ${\dot{Q}}_{perf}$: rate of heat dissipated by blood perfusion

• ${\dot{Q}}_{latent}$: rate of heat dissipated by latent heat loss through the respiration air flow, where the convective component is negligible in the inspiration thermal signal, due to outflow air with a temperature equal to the tissue temperature [20,27].

The convective heat transfer ${\dot{Q}}_{conv}$ of air flow over the inner lining of the nasal cavity and mucosal patches, where this convection is described by Newton's law of cooling, means that the rate of heat loss of a body (nasal cavity) is proportional to the difference in temperature between the body and its surroundings. Therefore, the rate of convective heat transfer is given by

(4) $${\dot{Q}}_{conv}\left(t\right)=k\cdot A\cdot \left(T_{env}\left(t\right)-T_{nasal-muc}\left(t\right)\right)=-k\cdot A\cdot \Delta T\left(t\right)$$

where Q is the thermal energy in joules, k is the heat transfer coefficient, A is the surface area of the heat being transferred (internal surface area of the nasal cavity), T_*nasal-muc *_is the temperature of the nasal cavity tissue (≈32°C) and T_*env *_is the temperature of the environment; i.e. the temperature that is a suitable distance from the surface (inflow air) and ΔT(t) = T_*nasal-muc*_(t)-T_*env*_(t) is the time-dependent thermal gradient between the environment and the object [19,28]. The radiation heat transfer ${\dot{Q}}_{rad}$ at the nostrils region is equal to

(5) $${\dot{Q}}_{rad}\left(t\right)=\varepsilon \cdot \sigma \cdot A_{c}\cdot \left(T_{nasal}^{4}-T_{c}^{4}\right)$$

where ϵ is the emissivity of the nasal tissue, T_*nasal *_is the nasal tissue temperature, T_*c *_is the surrounding's temperature and A_*c *_is the nasal tissue area. Consequently, the change in overall heat transfer energy leads to a dynamic change of air temperature and blood perfusion throughout the respiration cycle; therefore, the thermal signature develops within this cycle [18,28]. For the blood perfusion heat transfer, the blood acts as a local distributed, scalar source (or sink) of energy with a magnitude equal to:

(6) $${\dot{Q}}_{perf}\left(t\right)=\varsigma \cdot \rho_{bl}\cdot C_{bl}\cdot \left(1-k\right)\cdot \left(T_{art}\left(t\right)-T_{tissue}\left(t\right)\right)$$

where ζ, ρ_*bl *_and *C*_*bl *_are the blood perfusion rate, density and specific heat, respectively; k < 1 is a factor accounting for the incomplete thermal equilibrium between blood and tissue; and T_*art *_and T_*tissue *_are the arterial blood and tissue temperatures, respectively. The variable IR signature approximation which applies calculation of the maximal thermal contrast index (MTCI), which is denoted as C(t), is expressed as the principal parameter in pulsed thermography [17,21,22]. Therefore, this contrast index C(t) can be defined as follows:

(7) $$C\left(t\right)=\alpha_{cal}\cdot \left(T_{d}\left(t\right)-T_{0}\left(t\right)\right)$$

where *T*_0 _is the temperature at initial time, where temperature is minimal and *T*_*d *_is the temperature at final time, when temperature is maximal and *α*_*cal *_is the thermal camera calibration coefficient, which is adjusted according to the clinical thermographic setting.

### IRTR signal wavelet analysis

Continous wavelet transform (CWT) as introduced in [26,27] was applied to the IRTR signals. Essentially, the Debauchies (Db-wavelet) function was used with three decomposition levels [29]. The Db-wavelet was chosen instead of other functions (such as Haar, Biorthogonal and Morlet wavelets) because the Db-transformation is known to provide stable and accurate decomposition results for biomedical signals [26,29]. Thus, the thermal variation function f(t) is transformed as follows:

(8) $$CWT\left(a,b\right)=\frac{1}{\sqrt{C_{\Psi }}}\frac{1}{\sqrt{a}}\int_{-\infty }^{\infty }\Psi *\frac{t-b}{b}\cdot f\left(t\right)dt$$

where

(9) $$C_{\Psi }=\int_{-\infty }^{\infty }\frac{\Psi \left(\omega \right)}{\omega }d\omega <\infty$$

Furthermore, the Fourier transform of the wavelet function Ψ(t) is given by:

(10) $$\Psi \left(\omega \right)=\int_{-\infty }^{\infty }\Psi \left(t\right)exp\left(-i\omega t\right)dt$$

Eq. (10) implies that Ψ(*ω*) = 0 provided that *ω = 0 *and the dot symmetry condition is met, otherwise it is equal to 1 [30]. The function Ψ(t) is called the "mother wavelet". By shifting in time and dilating or compressing this function in frequency domain, one obtains a set of self-similar functions

(11) $$\Psi \left(a,b\right)\left(t\right)=\Psi \left(\frac{t-b}{a}\right)$$

where a ≈ *ω*^-1 ^is the scale that provides dilating or compressing, (b) is a time shift, and (t) is time. In contrast to discrete wavelet transform (DWT), large scales in CWT relate to the coarse representation of a signal, whereas small scales constitute its fine details [26,28,29]. Therefore, the definition of CWT entropy (WE) for an IRTR signal is:

(12) $$WE=-\underset{a}{\sum }p\left(a\right)log2p\left(a\right)$$

where p(a) is the probability distribution of IRTR signal at level (a). Therefore, the distribution can be approximated as:

(13) $$p\left(a\right)=\frac{E\left(a\right)}{E_{total}}$$

where E(a) is wavelet energy at level (a) calculated by

(14) $$E\left(a\right)=\underset{b}{\sum }|WT{|}^{2}\left(a,b\right)$$

where WT represents an orthonormal basis for wavelet transformation. Therefore, the total signal energy (E_*total*_) is

(15) $$E_{total}=\underset{a}{\sum }E\left(a\right)$$

**Figure 7:**
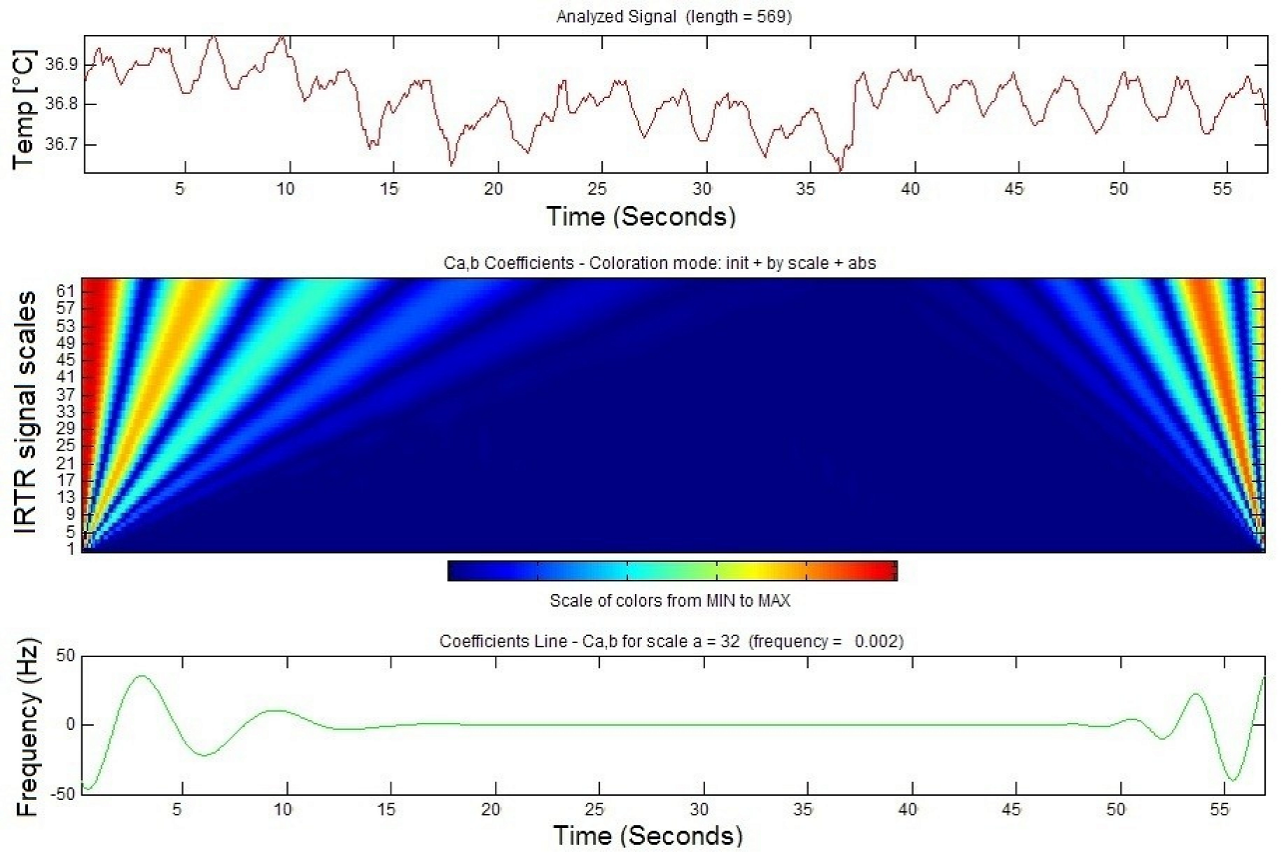
**IRTR signal wavelet analysis**. Wavelet analysis of the IRTR signal extracted from the region of the nostrils of a neonate during a one-minute interval.

Figure 7 illustrates the continous wavelet analysis of the IRTR signal extracted from ROI defined over the neonate nostrils, where the analysis performed over one minute interval.

### Neonatal respiration monitoring in the NICU

**Figure 8:**
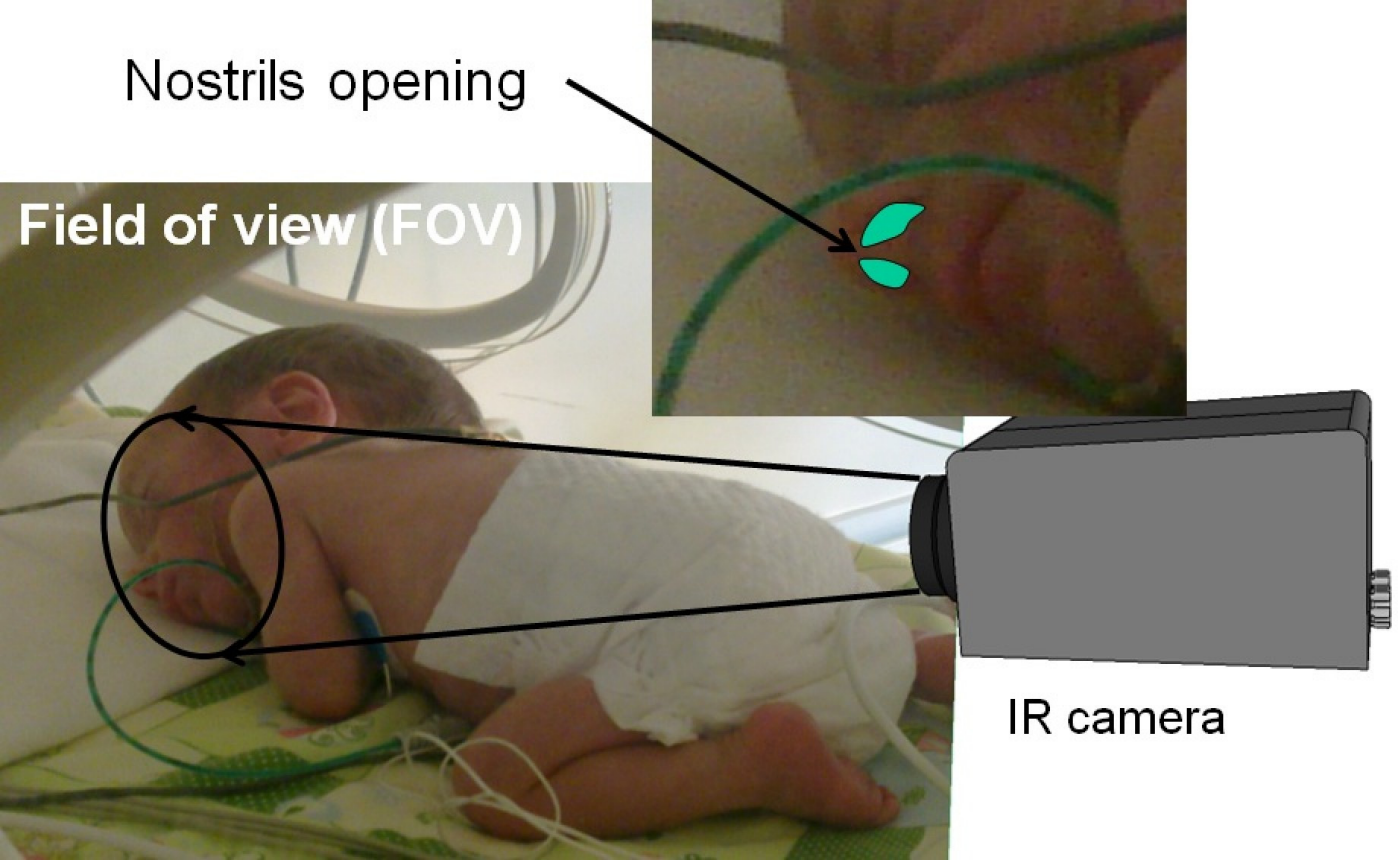
**IRTR camera setting with field of view (FOV) and region of interest (ROI) over neonate's nostrils**. Infrared camera setting for detection of neonatal respiration activity, showing the region of interest around the neonate's nostrils.

**Figure 9:**
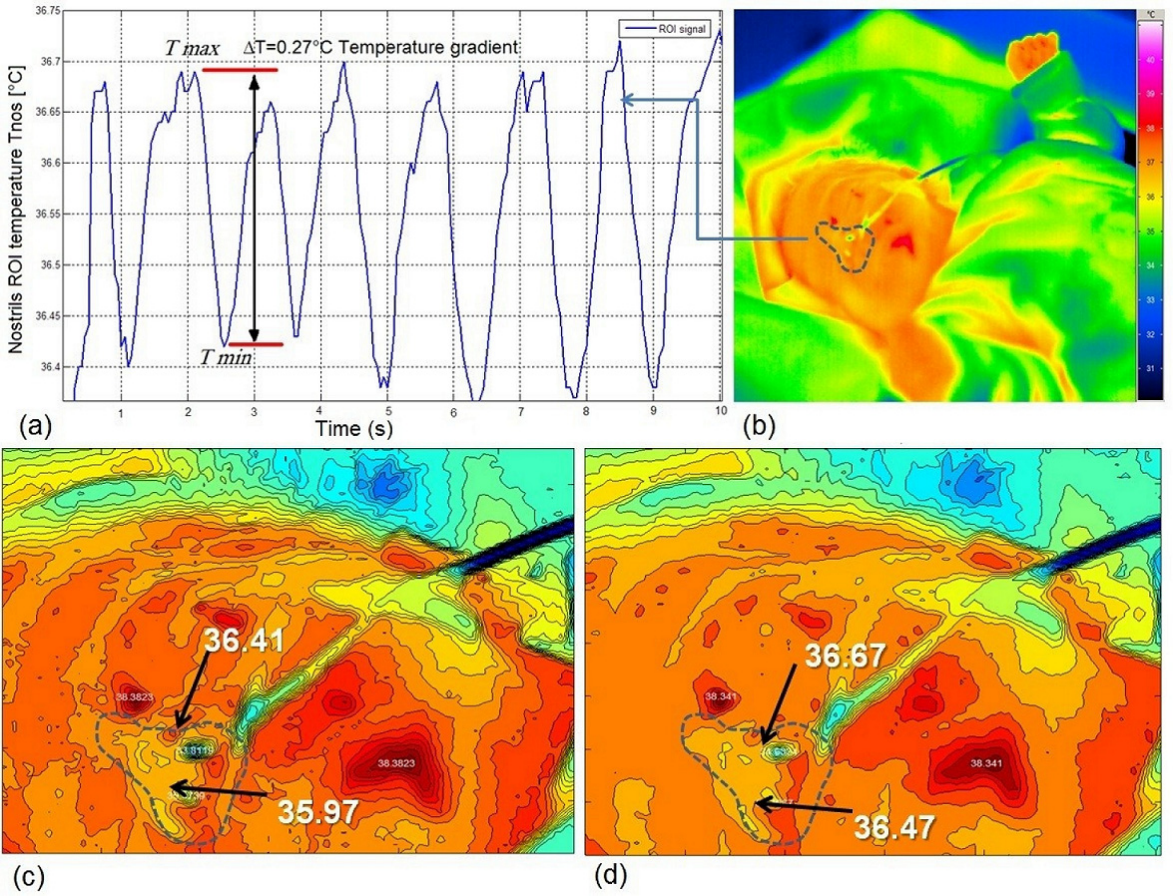
**IRTR image over neonate's nostrils**. Top: Neonatal infrared thermographic image with (a) IRTR signal with *δ*T = 0.27°C between inspiration and expiration (b) ROI located over the nostrils from which signal in (a) derived. Bottom: Thermal contour plot of the thermography clearly showing the thermal signature over this region at (c) inspiration phase starting and (d) at the expiration phase starting.

**Figure 10:**
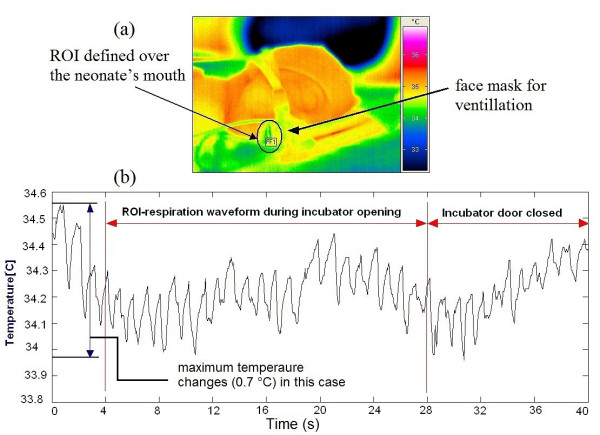
**IRTR signal of defined ROI temperature trend over neonate's mouth**. (a) Neonatal infrared thermographic imaging inside a neonatal incubator with the ROI around the mouth opening, (b) Fluctuating temperature trend of the neonate during normal activities of daily care.

**Figure 11:**
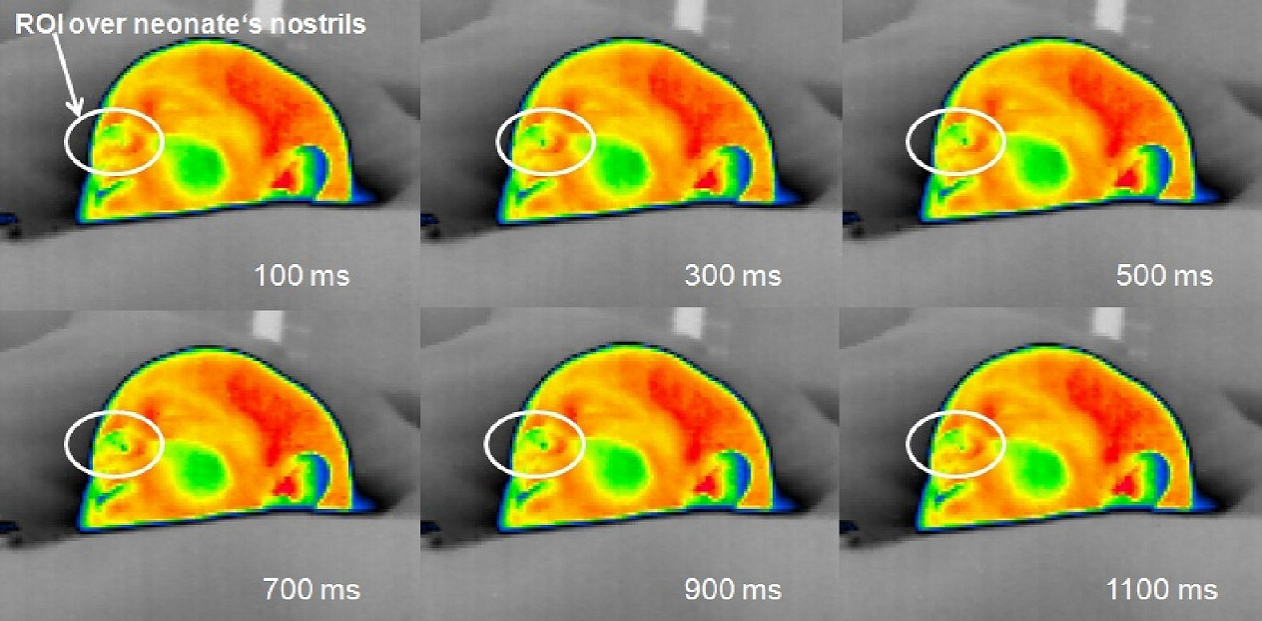
**Series of IRTR image during open care procedure**. Images of the IRTR signal detection process in the neonate. The sequence indicates tracking of one respiration cycle for 1100 ms; this interval is not fixed over the measurement period of time, but varies according to the physiological and clinical status of the neonate.

To the authors' knowledge, this is the first time that IRTR signal analysis has been applied to monitor neonates. Note that special clinical challenges are present because measurements take place in the NICU while patients may be highly vulnerable and physiologically unstable. Therefore, the configuration of the measurement setup should be such that it interferes in no way with any routine care procedures for the neonate. In some of the measurements, the ROI was taken over the baby's mouth, in order to quantify and detect the heat transfer pattern through the respiration phases [31]. This occurs mainly in CPAP or mechanical ventilation, or due to the inability to directly image the nostrils. However, in most spontaneously breathing neonates we focused on the nasal region (Figure 8 and Figure 9). As a reference, we recorded other vital signs such as ECG, heart rate and respiration rate, derived from the bedside intensive care monitoring module [31,32]. The temperature time course detected from neonate's nostril region is shown in Figure 10 (b), for a female neonate wearing a face mask and an ROI defined around her mouth cared inside a convective incubator (note the superposed temperature drifts due to changes in the internal temperature of the incubator). In the temperature signal derived from IR thermography, a temperature change of 0.5°C to 0.85°C related to the respiration rate thermal signature was visible (see Figure 10). In addition, actions of the nurse (e.g. opening the incubator door, handling of the baby) also influenced the temperature profile. Another important result is the reflection pattern of the air-jet stream on the reflective IR material (made of cotton) around the baby's face; when the baby exhaled air, the IR reflection signature can be clearly seen with this domain (although this is not quantitative but qualitative by nature). Figure 9 (top) illustrates a neonate placed under an IR radiant warmer with ROI defined over his nostrils with a good detected IRTR signature. In our study, most measurements were performed with newborn infants being subject to IR radiant warming therapy (see Figure 11). We noted that the process of IRTR signal detection in the neonate is more complex than in adults due to the following problems:

1. small air-flow jet in the neonatal respiration cycle and small lung volume

2. the possibility that part of neonatal IRTR signature located at other infrared spectral band, in which the current camera system is unable to detect the full variation in thermal energy

3. the geometry of the nasal aperture, i.e. the inner surface area of the nasal aperture (nostrils), is small (about 0.08 cm^2 ^as compared with about 1.07 cm^2 ^in the average adult).

4. the small amount of mucous secretion from the nasal cavity compared to adults

5. low humidity level in the nostrils region of the neonate

In fact, all factors mentioned above may reduce the amplitude of the IRTR signal. Therefore, some remaining problems need to be addressed in future investigations and further improvements should be performed taking into account the following: a) extending patient population (number of neonates considered in the study) to make more quantitative analysis, b) using more precise IR detector with high temperature resolution.

## Results and Discussion

**Figure 12:**
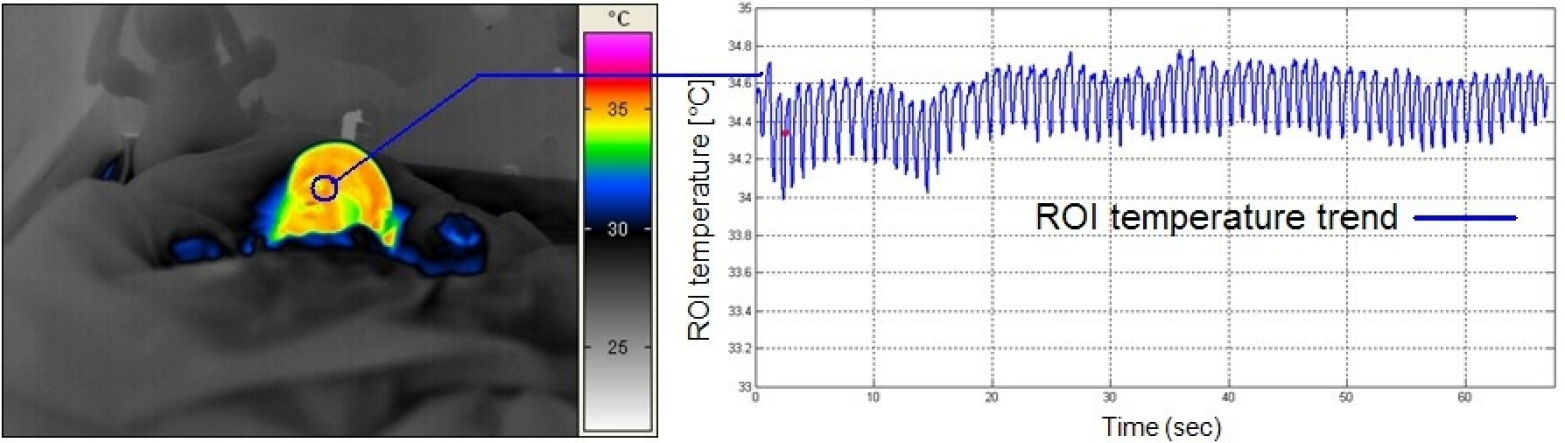
**IRTR signal of neonate under open therapy (IR radiant warmer)**. (Left) Neonatal respiration monitoring acquired from a newborn infant under the intensive open-care system, indicating that the respiration thermal signature is difficult to detect unless there is a good calibration and image zooming functions to identify temperature variation during the inspiration-expiration phases. (Right) Time graph of the ROI temperature profile over the nasal region, extracted and recorded for about 60 seconds.

In summary, the results presented here indicate that the IRTR thermal signature detection may be included into the future neonatal monitoring modalities. At present, the results acquired during IRTR measurement are not fully categorized and need more reliable measurement protocols. Additionally, the neonatal IRTR measurement are not correlated with a classical reference respiration sensing method, i.e. a thermistor that is fixed above the upper lip directly in front of the nares with the danger of inducing apnoeas because of upper airway obstruction. To overcome this problem, the neonate's respiration rate was manually registered from the bedside monitor. The disadvantage is that there is no information about the quality of breathing. The results from this experiment have shown clear changes in temperature over the nasal region and also a difference during inspiration and expiration. In addition, in some subjects averaging IRTR signals did not improve the signal quality and required further pre-procesing. By examining Figure 9, we notice that, for clinically stable infants (i.e. under IR radiant warmer), the IRTR signal magnitude is relatively higher than that of neonates cared inside convective incubator units (see Additional file 1: Neonatal IRTR video sample 1). Moreover, while changes in convective heat transfer due to respiration in adults are easily observed using an IR camera, respiration monitoring in the preterm infant remains a challenge due to a much smaller breathing temperature variation (see Figure 10 and Figure 12), and to other complex interactions (e.g. CPAP with face masks or prongs, mechanical ventilation, head rotation, motion artifacts, etc.) (see Figure 9) [26]. In addition, the detected IRTR signature depend also on the viewing angle set for the IR camera (see additional file 2: Neonatal IRTR video sample 2). Therefore, an optimal viewing angle will result in better quality of IRTR signature (see Figure 11). Regarding the standard reference measurement with IRTR signal, it seems there is also slight delay between the respiration signals derived from bedside ECG monitoring and the IRTR measurement (see Table 1). Therefore, by considering a lot of variation between each method, we state that the quality of the IRTR method approaches the established respiration activity recording using standard clinical measurement (e.g. ECG). Additionally, our finding regarding the small temperature difference over nostril region is already shown in Table. 1, were the maximum difference in temperature was about 0.66°C.

**Table 1:** Comparison of mean respiratory rate during 5-minute measurements derived from the IRTR method and from conventional ECG measurement

IRTR respiration derived parameters					
**Subj**	**RR (IRTR) [bpm]**	**RR (ECG) [bpm]**	**Minimum ROI temp [°C]**	**Maximum ROI temp [°C]**	

1	42.50	40.36	32.23	32.85	

2	44.25	42.60	32.11	32.77	

3	39.40	38.60	33.26	33.49	

4	45.14	45.20	32.18	32.45	

5	53.32	52.09	32.31	32.85	

In conclusion, whereas respiration jet and nostril temperature monitoring has been applied to adult volunteers, in this work IR thermography was shown to allow non-contact respiratory monitoring in neonates inside the NICU. Physically, the work is based on changes of convective heat transfer at the infra-nasal region, induced by breathing in dedicated ROIs. Until now the method seems more effective in adults than in newborns, due to the larger lung volumes in adults. Both the IR imaging device and the method itself still face drift problems due to variation in background temperatures. This requires improvements in image processing and boundary detection of the nasal region separated from the rest of the imaging scenario (e.g. incubator internal wall, mattress, and other facial regions). Moreover, the presented results are preliminary and need further studies in a larger number of neonates and under different care setups. The mathematical method needs further improvement, such as automatic ROI definition and automatic calibration. Furthermore, the IRTR monitoring may assist in the estimation of a possible temperature loss as a part of thermoregulation, and may be also considered as a first step to evaluate non-invasive respiratory behaviour of premature infants [33]. In comparison to the ECG derived respiration rate, the IRTR signal is correlated to this acquired signal from bedside monitor, while there is a slight difference in respiration rate estimated from each method (see Table 1). The main impediments to high resolution IRTR signature detection are the IR camera physical coverage and the thermal detector's resolution. In spite of the calibration mechanism in modern thermal cameras, most of medical IR imaging setups face calibration drift; and this needs enhancement in order to avoid erroneous measurement. To deal with this problem, the proposed solution is based on a virtual sensing mechanism to track the ROI over a defined anatomical part. Moreover, the information on intensity was extracted and transformed into a corresponding color-coded space of IR thermographic images.

## Conclusions

However, despite the limited number of measurements, these preliminary results provide a good basis for further investigation of the neonatal thermal respiration signature. More studies performed under standardized clinical conditions are needed, so that the method can be applied to examine the symmetrical pattern of the IRTR signature. Moreover, clinical investigations should explore a range of upper respiratory tract diseases. Furthermore, this method may be an effective quantitative technique to measure the nasal symmetrical air-flow pattern in preterm infants. This possibly can give information about the depth and the frequency of each breath cycle to get an early sign of changes in the infant's behavior. Also, the temperature difference up to 0.66°C can be interpreted as a part of the infant's thermoregulation and gives an interference of heat loss through expiration. Despite the remaining problems, the authors feel that the presented technique is a promising and effective step toward establishing cable-free monitoring of infants under intensive care conditions.

## Statement of consent

An oral consent was gained from the parents of the patient for publication of images and related files.

## Competing interests

The authors declare that they have no competing interests.

## Authors' contributions

AK conducted the experimental work and analysis of the thermography data, in addition he wrote the article (technical part) together with KH (clinical part). KJ and KH performed the clinical study at the Neonatal Intensive Care Unit (NICU). Prof. TO and Prof. SL supervised and coordinated the whole clinical and analytic work of this paper and carefully revised the paper. All authors read and approved the final manuscript.

## Supplementary Material

Additional file 1 **Neonatal IRTR video sample 1 - Neonatal respiration detection with IR thermography-Video 1**. This file is an audivisual file which illustrates the infrared thermography frame-sequence for detecting respiration thermal siganture from the nostrils region. This video file is taken through the preliminary clinical study on neonates cared in radiant warmer (open therapy). Click here for file

Additional file 2 **Neonatal IRTR video sample 2 -- Neonatal respoiration detection with IR thermography-Video 2**. This file is an audivisual file which illustrates the infrared thermography frame-sequence for detecting respiration thermal siganture from the nostrils region. This video file is taken through the preliminary clinical study on neonates cared inside convective incubator system (closed therapy). Click here for file
